# The Work of the Metropolitan Asylums Board.—II

**Published:** 1902-01-11

**Authors:** 


### The Institutional Workshop.

### THE WORK OF THE METROPOLITAN ASYLUMS BOARD.—II.

By the Author of "The Hospital Commissariat."

When the Metropolitan Asylums Board commenced its work in 1868 the estimated population of London had reached 3,000,000. Of this number 165,000, or 53 per 1,000, were in receipt of parochial relief, either indoor or outdoor, on the last day of January, and to these must be added about 1,000 casual paupers or vagrants. These figures do not, of course, give the total number of those chronically liable to need parochial assistance, but they may be regarded as the minimum which the Board had in view when considering the accommodation necessary. Roughly speaking, isolation hospitals were to be provided for any cases of infectious disease which might arise in a pauper population of some 200,000 people. The immediate difficulty in the way of the Board, and one which the lapse of 34 years has only intensified, was that of procuring sites for the erection of their hospitals. The first sites obtained were at Hampstead, Homerton, and Stockwell, and at Hampstead the first hospital of the board—a temporary structure—was erected in 1869, to receive cases of low fever, then very prevalent.

In 1870 and 1871 permanent buildings for the reception of fever and small-pox patients were erected at Stockwell and Homerton, and in the course of the next 14 months no fewer than 13,700 small-pox patients were treated in the new hospitals, in which 900 beds were then available. It was 20 years before the accommodation of the Board hospitals was strained to anything like the same extent again, and even in 1899, when the number of patients was higher than in any preceding year, and the number of beds available had been raised to 6,500, the total number of patients treated in all the 13 hospitals was less than double the number received during the great small-pox epidemic.

Two points were made indisputably clear by the events of 1871-2 after the immediate pressure and panic had passed away. In the first place the old definition of the duties of the Board, by which they were invited to the care of "poor persons chargeable to some union or parish," was found to have entirely collapsed. When the status of the patients was looked into it was found that only about 10 per cent. consisted of those for whom the hospitals had been originally built. No fewer than 90 per cent. were drawn from classes of independent citizens, to whom the brand of pauperism might have been imagined to be more peculiarly obnoxious than even the small-pox itself. It may fairly be claimed, we think, that the work of the voluntary hospitals of London is here to be seen.

The new hospitals were built and conducted on the lines which were already familiar and in which the public had learnt full confidence. The Poor-law stigma was visible merely in the introductory formalities and rapidly fell more and more into the background. The accommodation and treatment were all modelled on the voluntary hospital, and as the recovered patients began to come back with the tale of their experiences, public reliance became every day more firmly placed in the aid afforded.

There has been much terrible experience of the deadly determination of the poor in times of pestilence and panic to die in their own way at their own homes, and it is no trifling testimony to the extent to which the voluntary hospitals of London have won the hearts of the suffering poor, that isolation hospitals, altogether new in their experience, should have gained as they did immediate acceptance.

The public advantages to be gained by the immediate isolation of persons in all classes who would accept the accommodation of the Board hospitals were so great, that in 1879 an Act was passed to facilitate the Board in recovering the expenses of maintenance for any person, not a pauper, suffering from any dangerous infectious disorder, who should have been received in their hospitals. At the same time an ambulance service was commenced.

The general use of the Board hospitals was further encouraged by the facilities given by the parochial authorities to the removal of patients suffering from fever or small-pox in the ambulances of the Asylums Board at the request of the medical officer of health. The order of the relieving officer under which the patient was placed in the category of a pauper, came to be supplied after his removal as a mere formality, and the removal of all electoral disabilities from the receipt of sick aid completed the work.

By the time another epidemic was sweeping over London—that of scarlet fever in 1887—the hospitals of the Asylums Board were established as the recognised isolation centres for all who could not command the luxury of trained nursing and effectual isolation in their own homes.

Largely and freely as these hospitals were then resorted to, there were still dangerous delays in getting the order of the medical officer of health for the removal of patients in crowded quarters, and there were still instances in which, from ignorance, indifference, or laziness, outbreaks of fever were concealed by the relatives of the patient.

In 1889 the Infectious Disease Notification Act made it compulsory to inform the Medical Officer of Health immediately on the outbreak, in any house, of small-pox, diphtheria, enteric, typhus, or other infectious fevers. At the same time the order of the relieving officer, before or after admission to the hospitals of the Board, was rendered unnecessary, and the certificate of any duly qualified medical practitioner was substituted. This Act has completed the chain of events which has placed practically the whole population of London under the administration of the Board. Even the rich man who may desire to be removed to a nursing home or paying hospital for the treatment of infectious disease, may have the ambulance of the Metropolitan Asylums Board at his disposal; and the dreams of democracy seem to be very nearly translated into fact by the strange bedfellows with which fever may nowadays make a man acquainted.

On inquiring lately at one of the stations for observation maintained by the Board as to the status of the patients, the writer was informed that there were at present two patients in the wards; one was a visitor from the Hôtel Cecil, the other a person from a slum in Whitechapel.

We have said that two lessons were taught the Board by the small-pox epidemic of 1871-2: they discovered the popularity of their treatment from the point of view of the sick; they had also to learn the dangers of their hospitals to the public at large. It is not necessary to recall here the irresistible accumulation of facts which brought home to the minds of the authorities the impossibility of retaining small-pox hospitals in crowded neighbourhoods. It is sufficient to say that the danger once fully recognised was successfully combated. The small-pox patients were in 1881 removed to special hospital ships, and a special hospital camp was opened at Darenth for the reception of convalescent small-pox patients. The removal of patients is effected by steamers equipped as miniature hospitals for the purpose, and the arrangements for treating and isolating cases of small-pox in the metropolis have proved of great utility. The vigilance of the medical officers furnishes a pedigree, so far as it can be traced, of any case imported from without, giving a detailed account of each person infected by the original patient.

An important alteration in the administration of the Asylums Board was initiated in 1898. By order of the Local Government Board the management of the fever and small-pox hospitals was delegated to a Central Hospitals Committee.

